# Strategic Design and Multiperiod Optimization under Uncertainty of Solid Sorbent Direct Air Capture Supply Chains in Europe

**DOI:** 10.1021/acs.iecr.4c04040

**Published:** 2025-03-03

**Authors:** Daniel Crîstiu, Fengqi You, Federico d’Amore, Fabrizio Bezzo

**Affiliations:** † CAPE-Lab—Computer-Aided Process Engineering Laboratory, Department of Industrial Engineering, University of Padova, via Marzolo 9, 35131 Padova, Italy; ‡ Robert Frederick Smith School of Chemical and Biomolecular Engineering, Cornell University, Ithaca, New York 14853, United States

## Abstract

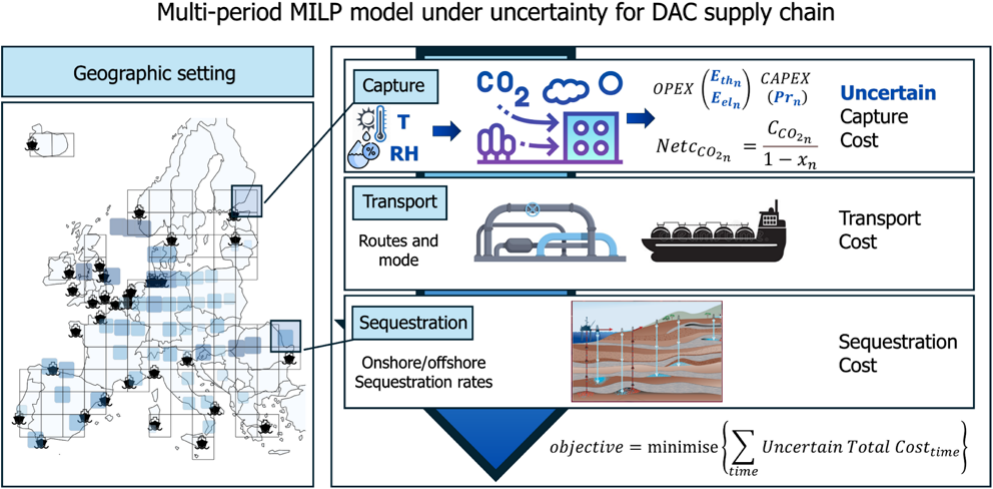

This study develops a multiperiod mixed-integer linear programming model for strategic planning of direct air capture (DAC) supply chains across Europe aiming at minimizing overall costs under uncertainty. DAC is pivotal for achieving net-zero targets and removing CO_2_ from the atmosphere to enable negative emissions. The optimization considers uncertainty in key parameters to ensure resilient decision-making. The model incorporates the influence of ambient air conditions on DAC performance, with temperature and humidity impacting productivity and energy consumption. Country-specific energy costs and greenhouse gas emission factors are accounted for, impacting the net cost of CO_2_ removal. Results indicate that with ambitious targets, technology learning curves, and renewable electricity transition, costs can fall to approximately 121 €/t CO_2_ by 2050, with 108 €/t attributed to capture costs. The findings highlight the importance of technological advancements and provide a systematic framework for policymakers to design resilient and cost-effective supply chains for large-scale deployment, positioning DAC as a potential decarbonization alternative for hard-to-abate emissions.

## 1 Introduction

Scenarios toward limiting global mean temperature increase below 1.5 °C have been extensively studied to explore pathways for achieving the 2015 Paris Agreement targets, and beyond. These scenarios, developed using integrated assessment models (IAMs), highlight the necessity of rapid transitions from traditional fossil fuels to large-scale, low-carbon energy supplies, reduced energy use, and significant carbon dioxide removal (CDR).^1−3^ The goal to limit global warming to well below 2 °C, and aiming for 1.5 °C, requires stringent limits on cumulative net CO_2_ emissions, which cannot be achieved without substantial CO_2_ removal.^4,5^ In fact, Luderer et al.^1^ used an ensemble of IAMs to explore the sector-level contributions to residual fossil CO_2_ emissions, finding that even with strong pre-2030 mitigation efforts and stringent long-term policies, significant CO_2_ removal (640–950 Gt CO_2_) will be necessary to achieve the 1.5 °C goal by 2100. Negative emissions technologies (NETs) play an important role in this context. Studies have shown that while there is growing research focused on the early stages of NET innovation, there is a lack of emphasis on the urgent actions required for large-scale deployment, being the key time frame for significant NETs deployment identified between 2030 and 2050.^6−8^ In fact, to align with the “well-below 2 °C” scenarios, it has been estimated that CDR deployment would need to grow exponentially, by 75–100% per year between 2020 and 2030.^5^ Furthermore, the recent IPCC sixth Assessment Report^8^ emphasized the fundamental role of CDR in climate mitigation strategies, highlighting its fundamental roles in accelerating near-term mitigation, achieving net-zero emissions, and generating net negative emissions to compensate for anthropogenic ones.^9,10^

Direct air capture (DAC) is a CDR technology that directly separates CO_2_ from the atmosphere. The importance of DAC in climate change mitigation is highlighted by its potential to address emissions from various dispersed and nonstationary sources, achieving negative emissions when combined with permanent storage,^11^ or utilization strategies in alternative chemicals and fuels,^12^ or long-lasting materials such as construction ones.^13^ Scaling up DAC to achieve gigatonne-level CO_2_ removal requires innovative approaches and significant mobilization of supply chains.^14^ To ensure the process results in negative emissions, challenges across process technology, techno-economic, and socio-political domains must be addressed.^15^ On the one hand, scaling up DAC involves investigating its technical performance, e.g., by understanding the properties of solid sorbents and liquid solvents, including mass transfer, heat transfer, and chemical kinetics. On the flip side, an effective DAC deployment also requires comprehensive policy support, including ensuring negative emissions, prioritizing long-term carbon storage, incentivising scale, codeveloping with capture, transport, and storage, phasing in a carbon price, coupling with renewables, harnessing hub deployment, maintaining separate targets, embracing certification and compliance, and fostering social acceptance.^16^ While learning-by-doing is crucial to drive down costs and improve material requirements, DAC costs are expected to fall, provided that technological learning is accelerated.^17^ Environmental trade-offs of large-scale DAC deployment must also be considered: while decarbonizing the electricity sector and improving DAC technology are key to avoid environmental problem-shifting, its optimal siting and planning of the energy requirements are critical to mitigate regional environmental impacts.^18^

Several studies have focused on predicting the cost of DAC technologies. For instance, assessments have indicated that initial DAC plants will be costly, but costs may decrease significantly with technological advancements and scaling.^16,17,19,20^ Sabatino et al.^21^ found that DAC technologies can provide high-purity CO_2_, with the solid-based process showing the best performance due to its lower exergy demand and higher productivity, while their capital cost was identified as the main cost driver, with all technologies potentially operating below 200 $/t CO_2_ under favorable conditions. Ozkan et al.^22^ highlighted that despite the growing number of DAC plants, the technology remains at an early stage of development, with current costs being 2–6 times higher than the threshold of 100 $/t CO_2_, and that costs depend significantly on the energy source used. Sendi et al.^23^ provided a comprehensive global techno-economic and environmental assessment DAC, highlighting that coupling DAC with thermal power plants, such as nuclear or natural gas with carbon capture, is the most cost and energy efficient option, aside renewables in specific regions with significant potential.

Innovative methodologies for technology cost forecasting based on empirical analysis of technical characteristics can provide probabilistic estimates of future DAC costs and offer insights into its economic feasibility of DAC.^24^ For instance, Lackner and Azarabadi^25^ explored cost reduction opportunities for DAC through learning-by-doing demonstrating that, similarly to other technologies such as solar photovoltaics, a significant initial capital investment could substantially reduce the cost of DAC to 100 $/t CO_2_. Young et al.^20^ indicated that DAC costs may decrease to 80–600 $/t CO_2_ in the long term at the gigaton scale. McQueen et al.^17^ reviewed various DAC technologies, emphasizing the importance of scaling up commercial technologies to drive down costs. Sievert et al.^19^ provided probabilistic estimates of future DAC costs, projecting about 374 $/t CO_2_ for solid sorbent DAC at 1 Gt/y cumulative capacity. Overall, the studies mentioned above provide valuable insights into the economics of DAC, but often lack of a comprehensive chain integration of CO_2_ transport and storage costs, as well as of a long-term strategic planning. Differently, Terlouw et al.^26^ focused on identifying suitable locations for grid-connected DAC supply chains in Europe by assessing its potential in relation to waste heat and renewable energy availability. However, this study used a static, current–time frame analysis and deterministic models with fixed input values, which could potentially lead to a lack of adaptability to changing conditions in the long term strategic planning. Additionally, that work did not include detailed analysis of the CO_2_ transport and storage stages, which are critical for understanding the full chain costs and logistics.

The objective of this study is to develop a multiperiod (2025 to 2050) mixed integer linear programming (MILP) framework for the optimal design under uncertainty of the DAC supply chain at the European level. The primary aim is to minimize the overall cost of the DAC supply chain, which includes capture, transport, and sequestration costs. This optimization considers the impact of ambient air conditions, energy costs, and greenhouse gas (GHG) emission factors specific to each European country. By considering site-specific ambient conditions and energy prices, the model identifies optimal locations for DAC installations and provides strategic insights for long-term planning and policy support to achieve net-zero targets. Furthermore, the model is used to analyze different scenarios, simulating various technological and economic pathways for DAC deployment. The productivity and energy consumption of DAC are obtained from the ambient air conditions, as modeled by Wiegner et al.,^27^ who investigated the performance of sorbent-based DAC processes under varying ambient conditions.

This study introduces several innovative aspects compared to previous methodologies in optimizing the deployment of DAC technologies in Europe. The following novel points can be identified:

- Unlike earlier studies, this work employs a multiperiod framework spanning from 2025 to 2050, allowing for dynamic and strategic planning over an extended time frame.
- The MILP approach accounts for uncertainty in key parameters such as contactor cost, energy consumption, and energy prices, enhancing the reliability and adaptability of the optimization model. The choice of optimization under uncertainty framework ensures resilience and performance under various future conditions, making the proposed DAC supply chain design effective under a wide range of scenarios.
- This study offers a detailed breakdown of the total cost into capture, transport, and sequestration costs, with capture costs further divided into capital and operative expenses as dependent on ambient conditions and energy costs.
- The spatial resolution adopted here discretises Europe into a 250 km grid, balancing geographic detail with computational feasibility, and evaluates both pipeline and ship transport options with multiple sizes for flexibility in the planning of CO_2_ logistics. Moreover, it accounts for climatic data for a comprehensive year-round operational assessment and includes country-wise GHG emission factors.

Summarizing, this study serves as a strategic tool for policymakers and stakeholders, emphasizing the potential for achieving net-zero targets through effective DAC deployment while addressing uncertainties and regional factors in detail for the large-scale setting of the European area.

## 2 Materials and Methods

This study aims at developing a multiperiod MILP framework to optimize a DAC supply chain from 2025 to 2050 at the European level, with the objective of minimizing the overall chain cost under uncertainty, i.e., by incorporating both capture, transport, and sequestration costs. This framework accounts for uncertainty in key parameters such as contactor cost, energy consumption, and energy prices. By considering the impact of ambient air conditions, energy costs, and GHG emission factors specific to each European country, the model identifies optimal locations for DAC installation and provides strategic insights for long-term planning to achieve the set CO_2_ removal targets under different scenarios.

**Figure 1:**
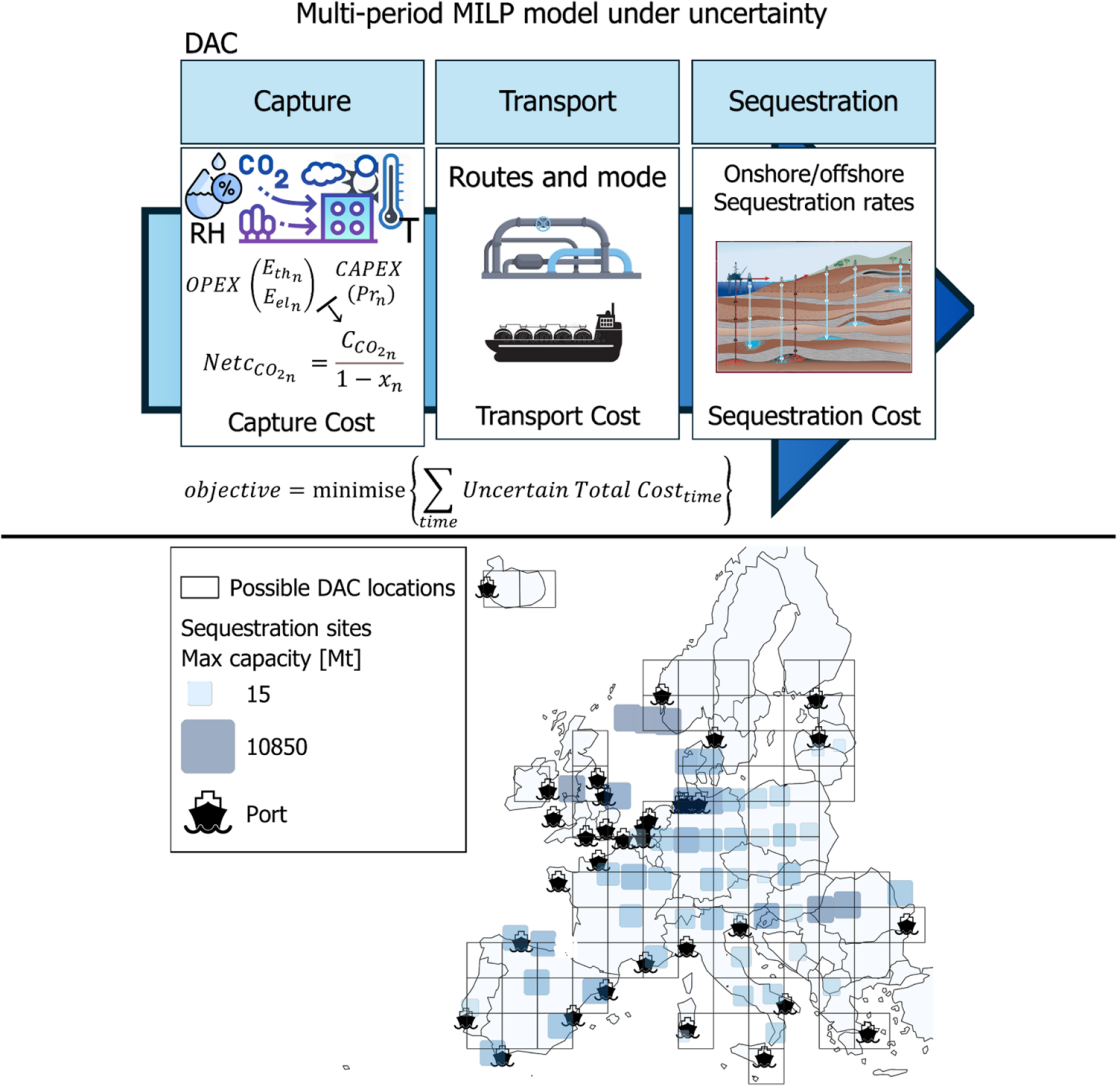
Graphical representation of the methodology and the geographic scope of the study, illustrating the potential locations for DAC installations and sequestration sites across Europe.

The geographical setting of this study covers the EU/EEA member states. Europe is divided into a grid of 250 km, resulting in 80 potential locations for DAC system installations (Figure 1). Additionally, 53 sequestration basins, both onshore and offshore, are considered across Europe, with CO_2_ transportation from capture to sequestration sites being possible via pipelines or ships. For each of the 80 potential DAC locations, seasonal average data (winter, spring, summer, autumn) for temperature and humidity were collected^28^ to determine the energy consumption and productivity of the solid sorbent DAC system via the correlations provided by Wiegner et al.,^27^ who based their work on Sabatino et al.^21^ The latter combined experimental data from four representative sorbents to study the average behavior without focusing on a specific material. Since that study did not report data related to temperatures below 5 °C, these were retrieved from Climeworks,^29^ considering an electricity consumption of 250 kWh/t CO_2_ and thermal energy consumption of 1750 kWh/t CO_2_. Productivity was averaged from the values of the other seasons.

The key decision variables include the location of DAC installation, the cumulative capacity of DAC at each location, the transport routes and modes, and the quantities of CO_2_ captured, transported, and sequestered. The spatially explicit features are described as nodes in the model through the set *n* of geographic locations comprising:

- *n* = {*n*_1–80_} ≡ location_*n*_, describing the possible DAC locations.
- *n* = {*z*_1–53_} ≡ seque_*n*_, describing 53 sequestration nodes, 3 of which are offshore.
- *n* = {port_1–33_} ≡ port_*n*_, describing 33 ports across Europe.

The flow rate discretisation necessary to determine the unitary transportation cost is described by the set *q* = {*q*_1–4_}, while the time-horizon is discretised through time periods *t* = {2025–2050} with a 5-year resolution (i.e., 6 time periods in total). To account for uncertainty in the model parameters, 10 000 scenarios are introduced using the set *s* = {scen_1–10 000_}. These scenarios represent different realizations of input parameters such as contactor cost, energy consumption, and energy prices. By incorporating a large number of scenarios, the model captures the variability and uncertainty inherent in these parameters. Each scenario represents a unique combination of input values, allowing the model to identify resilient solutions. This comprehensive exploration enhances the reliability and robustness of the optimization results. Data on the cost of electricity and thermal energy, as well as country-wise GHG grid emission factors, were collected for each centroid location of the square. These inputs, detailed in Supporting Information, contribute to the net capture cost calculation for the DAC solid sorbent. Also, the climatic data, the electric and thermal energy consumptions, the indirect CO_2_ emissions, and the details on the sequestration sites are reported in the Supporting Information.

### 2.1 Capture Stage

The solid sorbent DAC system utilized in this study employs a vacuum-temperature swing adsorption (VTSA) process for CO_2_ capture from ambient air, with sorbent regeneration occurring at 100 °C. This cyclic process includes four main steps: adsorption of CO_2_, preheating under vacuum to remove nitrogen, heating under vacuum to desorb CO_2_, and repressurization and cooling. Detailed information about the operation of this system can be found in the studies of Sabatino et al.^21^ and Wiegner et al.^27^ In particular, the latter^27^ was selected for describing the performance of the DAC unit due to its comprehensive data under varying conditions.

As for costs, in this work the expenditure for CO_2_ capture was computed as a function of three main variables: the air contactor cost (γ), and the electric and thermal energy cost, according to eq 1
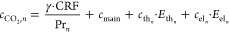
1where: γ represents the air contactor cost of one DAC module, with an average value of 236 000 €/module (Sabatino et al.^21^), Pr_*n*_ is the productivity in t/module/y in node *n*, *c*_main_ is the maintenance cost, set at 4% of the investment cost, *c*_th_*n*__,*c*_el_*n*__ are the heat and electricity prices^30,31^ in €/kWh in node *n*, respectively, and *E*_th_*n*__, *E*_el_*n*__ are the thermal and electrical energy consumption in kWh/t CO_2_ captured in node *n*. The chosen values for the air contactor cost cover a broad range of plant costs, from simple traditional columns^32^ to higher-cost full VTSA systems, comparable to the Hinwil Climeworks plant.^33^ The values used in this work align closely with Sievert et al.^19^ The capital recovery factor (CRF) is calculated as in eq 2
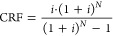
2where *i* is the discount rate of 0.1 and *N* is the plant lifetime, assumed to be 25 years.^27^

The net capture cost accounts for direct and indirect emissions resulting from the capture process, showing how much it would cost to remove a ton of CO_2_ from the air on a net life-cycle basis. These costs are related by a factor, *x*_*n*_, known as the take-back factor in node *n*, which here includes the energy related emissions associated with the DAC process.^34^ The net capture cost Net*c*_CO_2_,*n*_ [€/t CO_2_] in node *n* is then determined by eq 3(17)
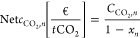
3where *x*_*n*_ is the take-back factor in node *n*, representing the amount of CO_2_ emitted from the process per tonne of CO_2_ captured from the air (Supporting Information).

### 2.2 Transport Stage

This study includes the possibility of transporting CO_2_ either via pipelines or ships. Offshore areas are divided into subsets, with transportation arcs classified as offshore based on the methodology of Crîstiu et al.^35^ Building on d’Amore et al.,^36^ this work allows for ship transportation to connect all potential sequestration sites across Europe. In particular, 33 major European ports were selected based on the gross weight of goods handled (Supporting Information).^37^

The unitary transportation cost for pipelines (UTC_pipe,*q*_ [€/t/km]) is categorized into four different pipeline sizes to reflect economies of scale associated with varying flow rates, using cost data from Rubin et al.^38^ As for offshore pipelines, the unitary transportation cost is increased by a factor of 1.5 compared to onshore infrastructures.^39^ Similarly, the unitary transportation cost for ships (*UTC*_ship,*q*_ [€/t/km]) is categorized to capture differences in transport efficiency and costs based on ship size, following the comprehensive methodology explained in d’Amore et al.,^40^ by incorporating the findings of IEAGHG^41^ to model ship transport costs through an array of slope (*UTC*_ship,sq_ [€/t/km]) and intercept (*UTC*_ship,*iq*_ [€/t of CO_2_]) coefficients, and by defining the same ranges of admissible flow rates as those for pipeline transport. The ship transport cost model assumes a shore-to-shore scenario and considers port-to-port transport with subsequent pipeline connections to reach the sequestration sites. Unitary transport costs are modeled considering prepressurized scenarios, as it is assumed that CO_2_ is compressed immediately after capture, with associated costs included in the capture stage. These detailed cost data, presented in Table 1, ensure a thorough analysis of the most cost-effective and efficient transportation routes for CO_2_ in Europe, considering both pipeline and ship transport options.

**Table 1:** Transport Ranges [*Q*_*q*_^min^, *Q*_*q*_^max^] [kt/y], Onshore and Offshore Unitary Transportation Costs through Pipeline UTC_*q*_^pipe^ [€/t/km] and Unitary Transportation Cost for Ship UTC_*q*_^ship^ [€/t/km]

flowrate discretisation			pipeline transportation cost		ship transportation cost
	range		UTC_*q*_^pipe^		UTC_*iq*_^ship^
size *q*	*Q*_*q*_^min^ [kt/y]	*Q*_*q*_^max^ [kt/y]	onshore [€/t/km]	offshore [€/t/km]	intercept [€/t/km]
*q*_1_	1	1500	0.054	1.5 × UTC^onshore^	12.911
*q*_2_	1500	2500	0.028		11.911
*q*_3_	2500	7500	0.016		7.911
*q*_4_	7500	100 000	0.010		7.911
					slope UTC_sq_^ship^ [€\t\km] 0.00609

### 2.3 Sequestration Stage

This study considers 54 possible CO_2_ sequestration sites, with their exact coordinates and maximum capacities detailed in the Supporting Information. The unitary sequestration costs (USC) are assumed equal to 7.2 €/t CO_2_ as reported by Rubin et al.^38^ For offshore storage, the cost for installing and operating injection wells is increased by a fixed parameter of 2.5.^42^ In our model, we analyze various sequestration injection rates over the designated time horizon to establish a realistic framework for sequestration capacity. This approach avoids dependence on a single site that may not handle the annual injection rates required. It also prevents the projection of unrealistically high rates that current technology cannot achieve. This allows to also prioritize sites capable of operation for at least 25 years, contributing to a practical and long-term approach to CO_2_ sequestration. Further details on the injection rates and the rationale behind these decisions will be provided subsequently.

## 3 Mathematical Formulation

### 3.1 Optimization under Uncertainty

The optimization under uncertainty of the DAC supply chain aims at finding optimal solutions under various scenarios or uncertainty in input parameters.^43^ The assessment incorporates uncertainty associated with price fluctuations, investment costs, energy prices, energy consumption, and productivity. The selection of uncertain parameters in this study was determined by their direct role in calculating the CO_2_ capture cost. As they inherently influence the overall cost of capture, all were included to ensure a thorough representation of uncertainty. To incorporate uncertainty, this study employs a Monte Carlo simulation to randomly sample from the distributions of uncertain parameters, for a total of 10 000 scenarios.^44^ Each scenario represents a different realization of the input parameters, and uncertain parameters include the contactor cost (γ), productivity (Pr_*n*_), thermal energy consumption (*E*_th,*n*_), electric energy consumption (*E*_el,*n*_), thermal energy cost (*c*_th,*n*_), and electric energy cost (*c*_el,*n*_). To incorporate uncertainty in these input parameters, this study utilizes either normal (Gaussian) or triangular distributions. The normal distribution is employed for parameters such as productivity, thermal and electric energy consumption, and thermal and electric energy costs. This distribution is appropriate when there is an assumption of symmetry around the mean value and well-understood variability: a deviation of 15% from nominal value is considered here. Conversely, the triangular distribution is used for parameters like contactor cost, where there is limited data available but some knowledge about the plausible range and the most likely value exists. The triangular distribution is characterized by a minimum, maximum, and most likely (mode) value. Accordingly, the CO_2_ capture cost of eq 3, in the uncertain formulation becomes as in eq 4
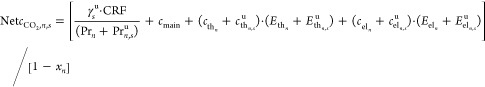
4where γ_s_^u^,Pr_*n*,*s*_^u^, *c*_th_*n*,*s*__^u^, *E*_th_*n*,*s*__^u^, *c*_el_*n*,*s*__^u^, *E*_el_*n*,*s*__^u^ represent the uncertain components of the contactor cost for scenario *s*, productivity, thermal energy cost, thermal energy consumption, electric energy cost, electric energy consumption all defined in node *n* and scenario *s*, respectively.

### 3.2 Multiperiod MILP Optimization

The objective function aims to minimize the total cost of the supply chain defined as the sum over all time periods *t*. In the formulation, uncertainty is incorporated into the objective function. The average-case objective function includes a penalty that averages the total cost over *N* = 10 000 different realizations of the uncertain parameters (scenarios *s*), represented by eq 5
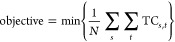
5where TC_*s,t*_ represent the total cost of the supply chain in scenario *s*, of period *t*. Total Cost of eq 6 is obtained by the contribution of the costs associated with the three stages of the SC, namely Total Capture Cost of scenario *s* in period *t* TCC_*s,t*_ [€/y], Total Transportation Cost in period *t* TTC_*t*_ [€/y], and Total Sequestration Cost in period *t* TSC_*t*_ [€/y]
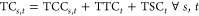
6TCC_*s*,*t*_ is the component of the objective function influenced by uncertainty, therefore it is defined in eq 7 on the set of scenarios *s*, and computed on the basis of the annual captured CO_2_ flow rate IN_*n*,*t*_ [t/y] in the location node *n*, in period *t*, representing the CO_2_ inlet to the transport stage, and Net*c*_CO_2_,*n*,*s*_ [€/t] the node-specific net CO_2_ capture cost in node *n* and scenario *s* defined in eq 4
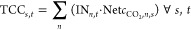
7The captured CO_2_ flowrate IN_*n*,*t*_ [t/y] in the location node *n*, in period *t* is constrained by the following equations (eqs 8 and 9) that represent the minimum and maximum bounds
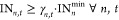
8
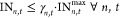
9where γ_*n*,*t*_ is a binary variable that takes the value of 1 if a DAC plant is installed in location *n* in period *t*, 0 otherwise; IN_*n*_^min^ represent the minimum installed capacity in location *n* and is 1 kt/y, while IN_*n*_^max^ represents the maximum cumulative capacity of DAC that can be installed in node *n*, and its values are presented subsequently. A continuity condition is enforced by a logical constraint in eq 10 to ensure that once a DAC plant is installed at a location node, it remains operational throughout the planning horizon
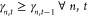
10To ensure that for each period the imposed target is met, the following constraint in eq 11 is added
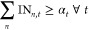
11where α_*t*_ [MtCO_2_/y] represents the target of period *t* in each scenario (see below).

TSC_*t*_ of eq 6 is computed based on the annual sequestered quantity of CO_2_ in sequestration node *n* and period *t* OUT_*n,t*_ [t/y], the unitary sequestration cost USC, and an additional cost for offshore sequestration USC_*n*_^offshore^ defined as a matrix that takes the value of Θ [= 2.5] if the sequestration site is located offshore, 1 otherwise, according to eq 12
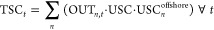
12However, OUT_*n*,*t*_ is constrained by the sequestration injection rate of each period *t* in eq 13
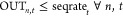
13
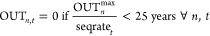
14where seqrate_*t*_ [MtCO_2_/y] is the injection rate of period *t* and is explained later. Equation 14 is constraining the use of sequestration sites that can be operated for at least 25 years; OUT_*n*_^max^ [Mt] represents the maximum capacity of the sequestration site (Supporting Information).

TTC_*t*_ of eq 6 includes the contribution of pipeline (TTC_*t*_^pipe^ [€/y]) and ship (TTC_*t*_^ship^ [€/y]) transportation costs according to eq 15
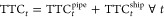
15Pipeline transport cost TTC_*t*_^pipe^ is calculated in eq 16 as follows
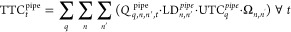
16where *Q*_*q,n,n*′*,t*_^pipe^ [tCO_2_/y] is the quantity transported with a pipeline of size *q*, between nodes *n* and *n*′, in period *t*, and is discretized through the following boundary constraints in eqs 17 and 18
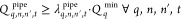
17
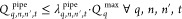
18λ_*q,n,n*′*,t*_^pipe^ is a binary variable that takes the value of 1 if a pipeline of size *q* is installed in between nodes *n* and *n*′, in period *t*, 0 otherwise, and *Q*_*q*_^min^ and *Q*_*q*_^max^ are the minimum and maximum flowrates for the different sizes of pipeline as presented in Table 1. The other components of eq 16 are UTC_*q*_^pipe^, which is the unitary transport cost via a pipeline of size *q*, and Ω_*n,n*′_ [= 1.5], which represents the additional cost of offshore pipeline installation. LD_*n,n*′_^pipe^ is the linear distance between nodes *n* and *n*′ and is calculated in eq 19 using the law of cosines as in the methodology presented in d’Amore and Bezzo,^45^ which is based on geographic coordinates *X*_*n*_ [rad] and *Y*_*n*_ [rad] of node *n* and the earth radius *R* [= 6372.795 km]. Transportation arcs are reduced by setting an upper bound LD_pipe_^max^ [= 260 km] that acts as a threshold distance to reduce the combinatorial connectivity among the nodes for the feasible transport routes as in eq 20
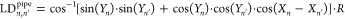
19
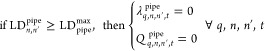
20A continuity constraint for pipeline transport is then needed to ensure that once a pipeline is installed, it remains operational throughout the planning horizon. Specifically, this constraint in eq 21 requires that if a pipeline is active in a given period, it must also be active in subsequent periods
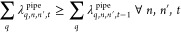
21Ship transport cost TTC_*t*_^ship^ of eq 15 is calculated through a linear regression as in eq 22
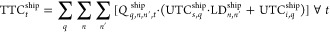
22where *Q*_*q*,*n*,*n*′,*t*_^ship^ [tCO_2_/y] is the quantity of size *q* transported with a ship, between nodes *n* and *n*′, LD_*n,n*′_^ship^ is the naval distance between ports *n* and *n*′,^46^ and UTC_*s,q*_^ship^ is the slope and UTC_*i,q*_^ship^ intercept coefficients presented in Table 1. Similarly to pipeline transportation, also the quantity transported via ship is discretised according to eqs 23 and 24
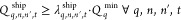
23
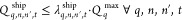
24λ_*q*,*n*,*n*′,*t*_^ship^ is the binary variable that takes the value of 1 if a quantity *q* is transported via ship between nodes *n* and *n*′ in period *t*.

The sum of the quantity transported with pipelines and ship gives the total transported quantity *Q*_*n*,*n*′,*t*_ of eq 25, which is then used to write the mass balance equation between nodes *n* and *n*′ in eq 26
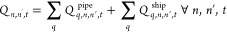
25
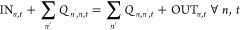
26

### 3.3 Scenarios

The growth rate of DAC technology has been derived from the European CO_2_ availability report for EU-27 members and the UK.^47^ However, the required capacities in each location are based on announced projects and data retrieved from DAC Coalition.^48^ Significant capacity increases are essential to meet the low and high DAC demand scenarios, reflecting the ambitious CO_2_ reduction targets set by the EU. Table 2 summarizes the annual capacity growth rates required to meet DAC demand in 2030, 2035, 2045, and 2050, starting from a base capacity of 1 MtCO_2_/y in 2025. The projected growth is supported by ongoing projects and technological advancements. In addition to this, the sequestration rate is an important factor. The global injection rate projected by the IPCC is between 3 and 10 Gt/y by 2050, with well-established costs for injection rates of 0.5–5 Mt/y.^49^ In the EU, an expansion of 80–300 Mt/y by 2050 has been announced. Table 2 shows the injection rate trajectory considered in this study, assuming a 10% annual increase from 2025 to 2050, starting with 5 Mt/y in 2025.^50^

**Table 2:** Growth Rate of Direct Air Capture Technology over the Period 2025–2050,^47^ and the Maximum Installed Capacity in One Location^48^

*t*		2025	2030	2035	2040	2045	2050
lowDAC	α_*t*_ [MtCO_2_/y]	1	10	56	116	182	281
	max location capacity In_*n,t*_^max^ [ktCO_2_/y]	50	267	1294	2000	3000	4500
highDAC	α_*t*_ [MtCO_2_/y]	1	10	87	234	359	442
	max location capacity In_*n*,*t*_^max^ [ktCO_2_/y]	50	267	1294	4000	5000	6000
sequestration injection rate seqrate_*t*_ [MtCO_2_/y]		5	8	13	20	34	54

The cumulative maximum capacity of one location^48^ assumes starting with a capacity of 50 kt CO_2_/y in 2025, to then scale up to 267 kt CO_2_/y by 2030, supported by current project announcements and technological advancements. By 2030–2035, this increases to 1294 kt CO_2_/y, reflecting a feasible expansion considering the accelerating pace of DAC technology. From 2035 to 2040, a 55% increase leads to 2000 kt CO_2_/y, accounting for the need of additional infrastructure. Continuing with a 50% increase from 2040 to 2045, DAC reaches 3000 kt CO_2_/y, maintaining the growth trajectory. Achieving the value of 4500 kt CO_2_/y by 2050 with another 50% increase stays within realistic boundaries, reflecting the expanded capacity with several projects aiming to capture large volumes. The assumptions made above are valid for the LowDAC demand scenario, while in the HighDAC one, from 2040 onward, the maximum location capacity is assumed proportional to the growth rate.

To evaluate the cost of DAC and understand its evolution over time, this study considers two scenarios, each with six subcases as presented in Table 3. These, denoted as S1.1–6 for LowDAC demand and S2.1–6 for HighDAC demand, examine various configurations of the supply chain

- S1.1 and S2.1 involve multiperiod optimization under uncertainty of the DAC supply chain, serving as the reference case.
- S1.2 and S2.2 allow only offshore sequestration for the captured CO_2_.
- S1.3 and S2.3 implement a 12% learning curve for DAC capture costs based on cumulative capacity.
- S1.4 and S2.4 assume DAC is fully electrified and powered exclusively by renewable energy.
- S1.5 and S2.5 combine the 12% learning curve with fully electrified DAC powered by renewable energy.
- S1.6 and S2.6 integrate the 12% learning curve, renewable energy, and restrict sequestration to offshore locations.

**Table 3:** Summary of the Analyzed Scenarios

scenario		multiperiod optimization under uncertainty	only offshore storage	learning curve	fully renewable energy input
lowDAC	S1.1	x			
	S1.2	x	x		
	S1.3	x		x	
	S1.4	x			x
	S1.5	x		x	x
	S1.6	x	x	x	x
highDAC	S2.1	x			
	S2.2	x	x		
	S2.3	x		x	
	S2.4	x			x
	S2.5	x		x	x
	S2.6	x	x	x	x

These scenarios help understanding the implications of different technological and economic pathways over DAC deployment. Differently, the learning curve approach reflects how costs decrease as cumulative capacity increases, with a fixed reduction rate of 12% for every doubling of capacity.^19^ In this analysis, the learning curve is applied to specific cumulative capacity milestones that reflect significant stages in the deployment of this technology (Table 2). These milestones start from an initial capacity of 1 Mt/y.

## 4 Results and Discussion

The optimization of the DAC supply chain was performed on a DELL Precision 7560 laptop with Intel(R) Core (TM) i7-11850H @ 2.50 GHz 2.50 GHz and 64 GB RAM. Specifically, the multiperiod MILP formulation under uncertainty was implemented in GAMS 43.3.0 and solved through CPLEX. The details of the optimization are as follows: number of blocks of variable: 33, single variables: 4.76 million, binary variables: 2.05 million, average solving time: 3.5 h, average optimality gap: ≤0.1%. Results are presented in terms of economic optimum for the two main analyzed scenarios, LowDAC and HighDAC.

### 4.1 LowDAC Scenario

**Figure 2:**
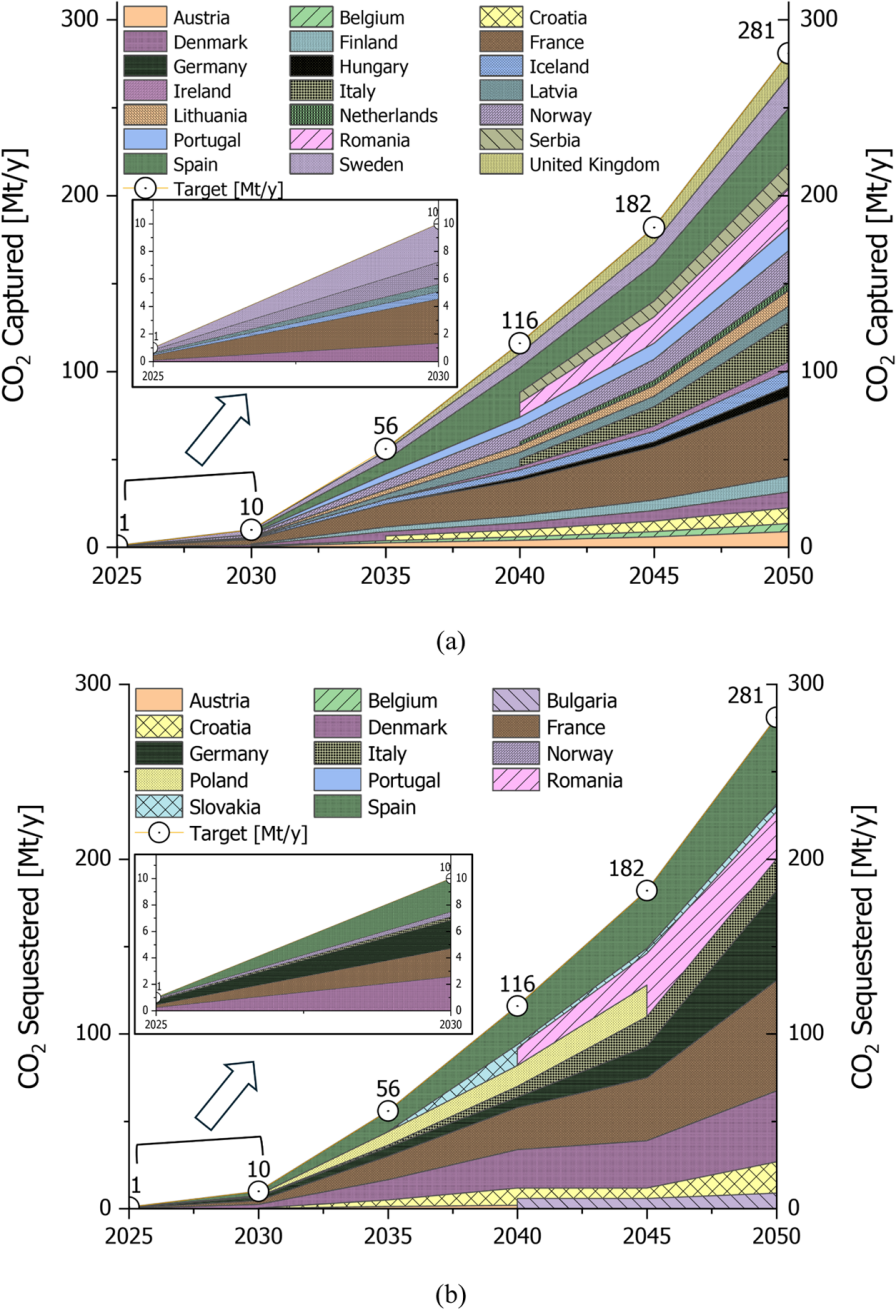
LowDAC scenario (S1.1) (a) CO_2_ captured quantities in European countries for different periods, (b) CO_2_ sequestered quantities in European countries for different periods. On both figures the target for each period is displayed.

The LowDAC scenario includes a series of subscenarios (S1.1–6) that explore different configurations and constraints for deploying DAC technology across Europe. These scenarios are considered to reflect a conservative approach to scaling DAC technology, considering factors such as sequestration options, cost reductions due to learning curves, and renewable energy integration. Figure 2 illustrates the optimal CO_2_ captured and sequestered quantities across European countries from 2025 to 2050 for the baseline subscenario S1.1. In particular, Figure 2a highlights significant contributions in terms of captured CO_2_ (starting from 2025) from France, Norway, Sweden, Denmark, Iceland, and Latvia. The optimization of these locations considers several key factors to minimize overall capture costs. Ambient air conditions, such as temperature and humidity, are important as they significantly impact the efficiency of solid sorbent DAC systems, affecting both productivity and energy consumption. Locations with air conditions that enhance DAC efficiency, such as low ambient temperatures and moderate-to-high humidity levels, are prioritized to ensure economical operation. The model does not account for extreme weather conditions, as it includes average annual weather data from Climate Copernicus.^28^ Another critical factor is the energy mix. The selected locations feature a low carbon footprint in their primary energy sources, which is essential for reducing the (indirect) carbon emissions associated with DAC operations. Utilizing low-emission energy or renewables helps to minimize the carbon impact of the capture process, and therefore lower the cost of the net capture CO_2_ through the DAC system. Proximity to sequestration sites is also considered to reduce transportation costs. Locations near sequestration sites help lower logistical expenses by minimizing the CO_2_ transport distance. Figure 2b shows the CO_2_ sequestered quantities and the data indicates that sequestration capacities align with the capture targets, with notable contributions from France, Germany, Norway, and Italy from 2025. This arrangement ensures an effective and efficient distribution of CO_2_ capture and sequestration efforts, aligning with the LowDAC scenario targets.

**Figure 3:**
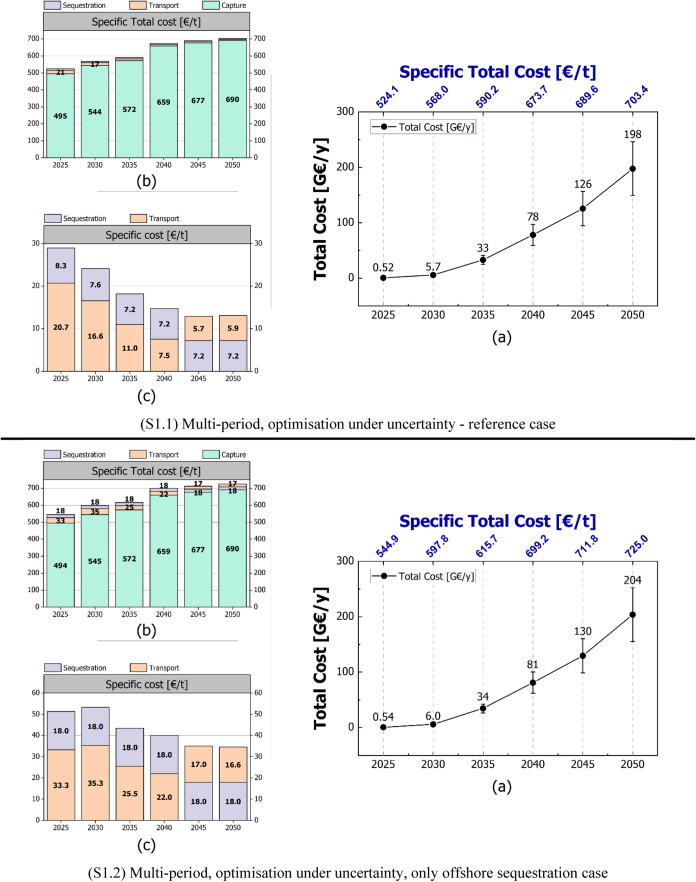
LowDAC scenario (S1.1) (a) uncertain total cost of the supply chain for different periods in [G€/y] and the corresponding specific total cost in [€/t], (b) specific total cost breakdown into specific capture cost, specific transport cost and specific sequestration cost in [€/t], (c) detailed view from (b) focusing on the specific cost of transport and sequestration stages; (S1.2) only offshore sequestration scenario with the corresponding total cost and specific total cost figures as mentioned in (S1.1).

**Figure 4:**
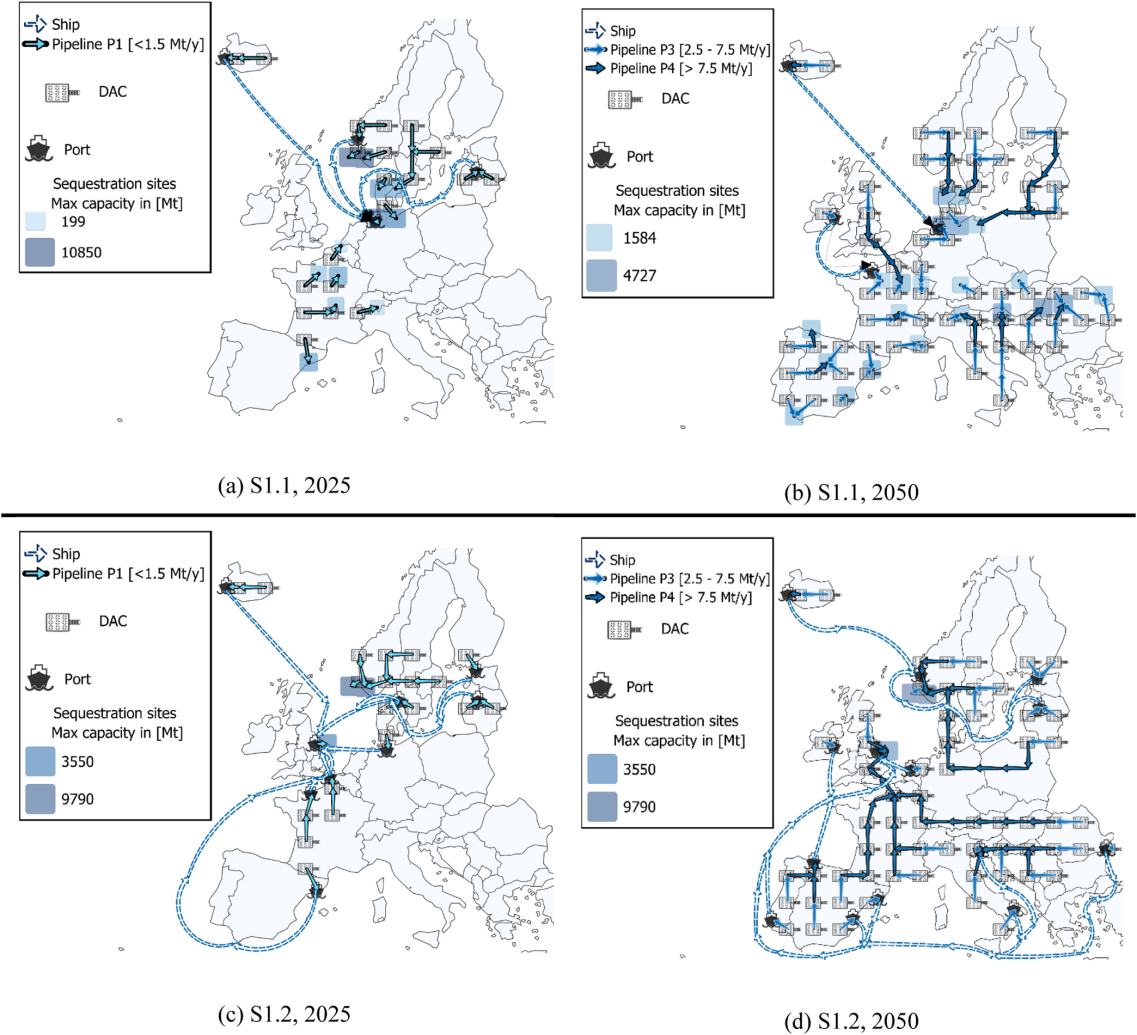
LowDAC Scenario Configurations: (a) supply chain configuration for the year 2025 in scenario S1.1, (b) supply chain configuration for the year 2050 in scenario S1.1, (c) supply chain configuration for the year 2025 in the offshore scenario (S1.2), (d) supply chain configuration for the year 2050 in the offshore scenario (S1.2).

Figure 3 provides a comprehensive analysis of the total and specific costs under uncertainty of the DAC supply chain for the LowDAC scenario, examining two cases: the reference case (S1.1) and the offshore sequestration case (S1.2). The total cost under uncertainty reflects the cumulative expenses of capture, transport, and sequestration across the entire supply chain. It emerges that for the reference case (S1.1), the uncertain total cost of the DAC supply chain increases over time, aligning with the scale-up of CO_2_ capture required to meet the decarbonization target. The specific total cost, which measures the cost per ton of CO_2_ captured, also increases over time as higher-cost locations for net CO_2_ removal are selected over time. This trend is better illustrated in Figure 4, where the supply chain configurations are presented. The assumptions for this scenario represent a pessimistic case with no cost reductions due to learning curves, constant energy mixes across all periods, and no changes in energy costs, nor economies of scale, or technological advancements. In 2025, the total cost of the supply chain is 0.54 G€/y, with a specific total cost of 524 €/t, increasing to 198 G€/y in 2050 and a specific total cost of 703 €/t. The specific capture cost constitutes the largest portion of the total cost, representing 94% of the specific total expenditure in 2025 (494 €/t out of 524 €/t) and 98% in 2050 (690 €/t out of 703 €/t), while transport and sequestration costs are relatively marginal contributions. Over time, the specific cost of sequestration decreases due to a shift to less expensive onshore sequestration options.

In the offshore sequestration case (S1.2), the total cost is higher compared to the reference case due to increased transport costs, as offshore sequestration sites are more expensive than onshore ones and need to be reached by long-distance shipping (Figure 4). The specific sequestration cost is notably higher in this scenario, highlighting the economic impact of limiting sequestration to offshore locations. However, the specific total cost only slightly increases, by approximately 3% from the reference case in 2025 and 2% in 2050. These results demonstrate the economic trade-offs involved in different DAC deployment strategies. Although restricting sequestration to offshore locations increases transport and sequestration costs, the overall impact on the specific total cost is relatively small. Therefore, offshore sequestration is not only a feasible option when onshore sites are limited or unavailable, but also when policy conditions are uncertain or evolving. As such, offshore options can offer a more stable alternative for CO_2_ storage.

Figure 4 illustrates the supply chain configurations for the reference case (S1.1) and the offshore sequestration case (S1.2) in 2025 and 2050. As in Figure 4a for the reference case (S1.1), in 2025 DAC installation is strategically located in countries that minimize the cost of net CO_2_ removal and are in the proximity of the sequestration sites. This optimal setup ensures efficient transportation routes, being the CO_2_ transported to the nearest sequestration site. For smaller quantities and longer distances, naval shipping is preferred over pipelines, as observed in Iceland and Latvia. While some CO_2_ is sequestered offshore in 2025, transporting it via ship to alternative onshore sites with lower sequestration costs proves then more cost-effective. In fact, by 2050, pipelines are mostly deployed, with the exception of Iceland and Ireland, where naval transport emerges still cost-competitive with respect to offshore pipelines. The optimal 2050 configuration includes exclusively onshore sequestration sites, prioritising those with higher total capacities. As for the offshore storage scenario (S1.2), it limits sequestration to offshore locations and relies more heavily on shipping to connect southern Europe with major offshore sequestration sites in Northwestern Europe. While this approach results in higher transportation costs, the overall impact on the total supply chain cost remains relatively small. Therefore, integrating offshore sequestration and ship transportation into the infrastructure can represent a feasible and effective strategy.

### 4.2 HighDAC Scenario

The HighDAC scenario explores a more ambitious approach to scaling DAC across Europe, considering higher growth rates and larger capacities than the LowDAC case. Initially, in the first two years, both scenarios have similar targets to meet. However, from the second year onward, the HighDAC scenario sets more ambitious targets, reflecting a higher growth rate in DAC capacity. This is why the supply chain configuration is only shown for 2050, as the early years are similar to the LowDAC scenario.

**Figure 5:**
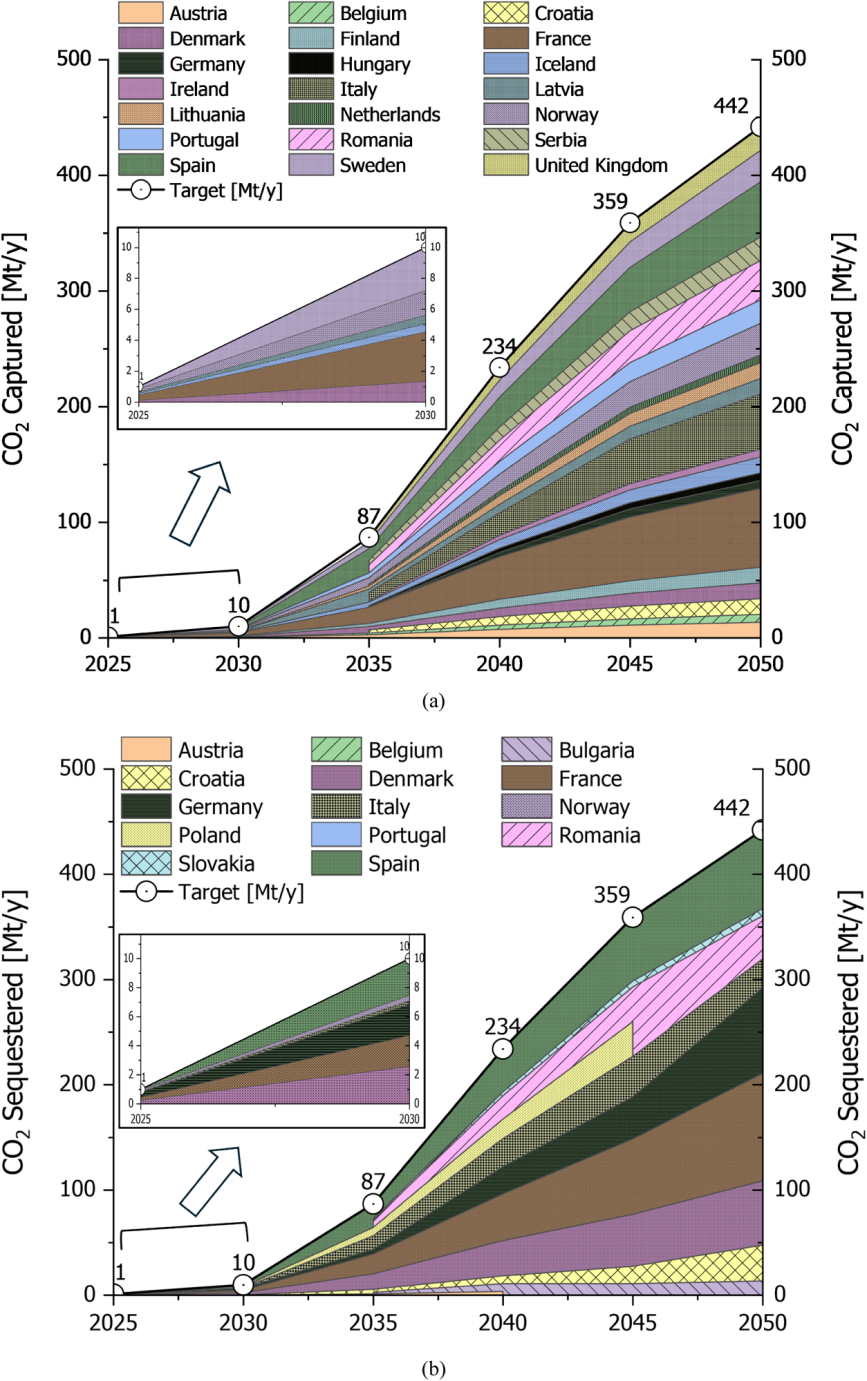
HighDAC scenario (S2.1) (a) CO_2_ captured quantities in European countries for different periods, (b) CO_2_ sequestered quantities in European countries for different periods. On both figures the target for each period is displayed.

**Figure 6:**
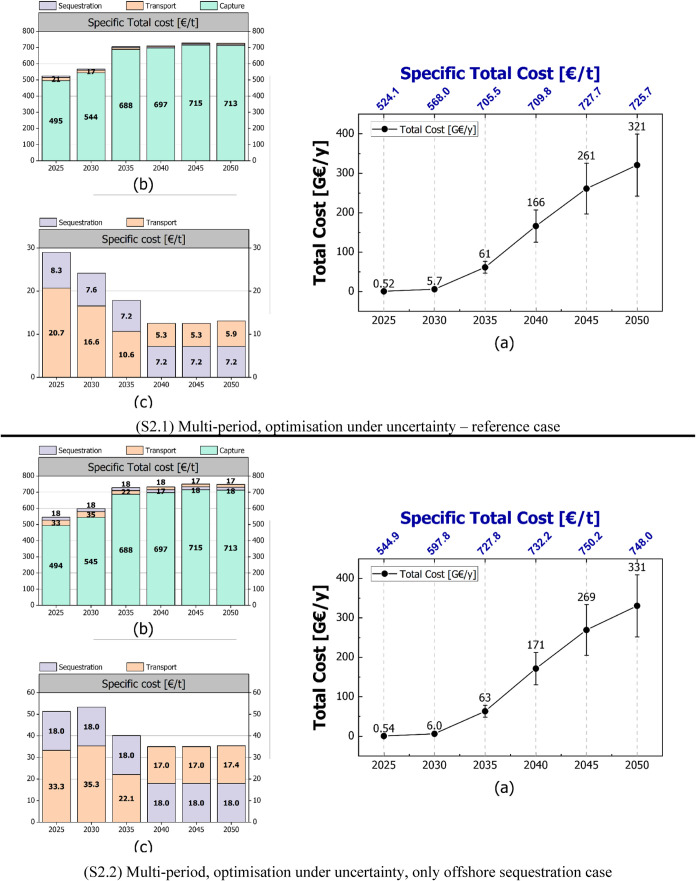
HighDAC scenario (S2.1) (a) uncertain total cost of the supply chain for different periods in [G€/y] and the corresponding specific total cost in [€/t], (b) specific total cost breakdown into specific capture cost, specific transport cost and specific sequestration cost in [€/t], (c) detailed view from (b) focusing on the specific cost of transport and sequestration stages; (S2.2) Only offshore sequestration scenario with the corresponding total cost and specific total cost figures as mentioned in (S2.1).

Figure 5 illustrates the projected quantities of CO_2_ captured (a) and sequestered (b) across various European countries from 2025 to 2050 for the HighDAC scenario (S2.1). The figures show substantial increases in CO_2_ capture and sequestration capacities over the years to meet the set targets compared to the corresponding LowDAC case. A detailed analysis of the uncertain total and specific costs of the DAC supply chain under the HighDAC scenario is given in Figure 6, by comparing the reference case (S2.1) and the offshore sequestration case (S2.2).

The general trend observed in Figure 6 is similar to that observed in the LowDAC scenario. The most pessimistic scenario (S2.1) assumes no cost reduction through economies of scale or learning curves, nor transition to renewables, resulting in an increase in specific total costs until a certain point. Notably, the specific total cost shows an initial increase to then flatten out. All possible locations are used for CO_2_ capture, with the exception of those that would result in a positive GHG emission factor due to indirect emissions, such as Poland and Estonia, or locations with very high GHG emission factors making the net cost of CO_2_ removal prohibitively high, such as Greece, Bulgaria, Serbia, and specific locations in Germany and Italy. When comparing the reference case (S2.1) with the offshore sequestration case (S2.2), the latter shows a marginally higher cost. Specifically, by 2050, the specific total cost for the offshore scenario results approximately 3% higher than the reference scenario, with 748 €/t compared to 726 €/t, respectively. This small increase indicates that offshore sequestration is a feasible option, adding only a limited financial burden to the overall DAC supply chain.

**Figure 7:**
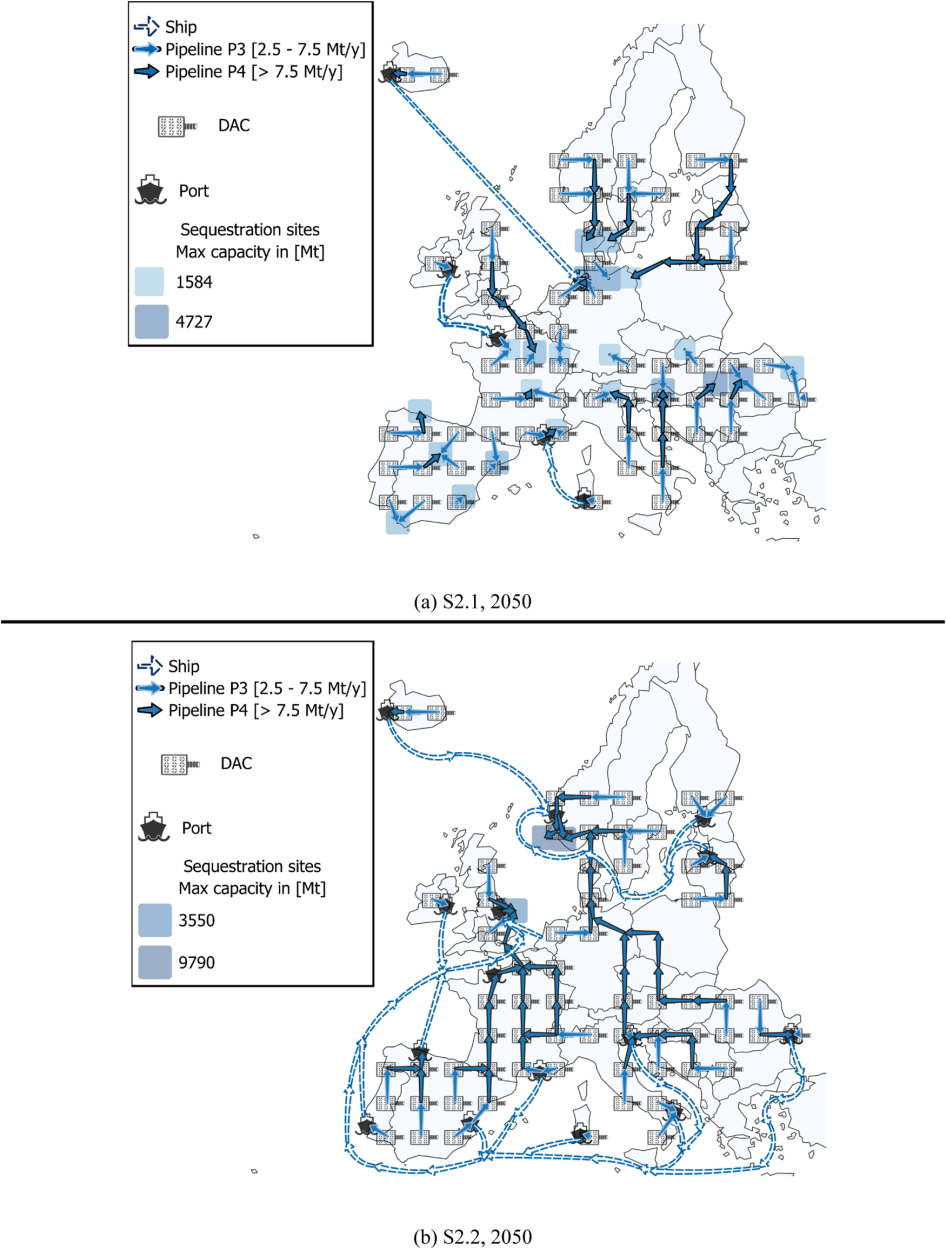
HighDAC scenario configurations: (a) supply chain configuration for the year 2050 in scenario S2.1, (b) supply chain configuration for the year 2050 in the offshore scenario (S2.2).

Figure 7 illustrates the supply chain configurations for the HighDAC scenario in 2050, comparing the reference case (S2.1) and the offshore sequestration case (S2.2). In particular, Figure 7a shows the configuration for the reference case and highlights the strategic placement of DAC installations and the predominant use of pipelines and (rarely) naval shipping to transport the CO_2_ to the sequestration sites. Differently, Figure 7b shows the chain configuration for the offshore scenario, which heavily relies on shipping to connect DAC installations in southern Europe with major offshore sequestration sites in Northwestern Europe. As for LowDAC cases, naval shipping is preferred over installing offshore pipelines due to its economic advantage over offshore transportation arcs.

### 4.3 Specific Total Costs Comparison

**Figure 8:**
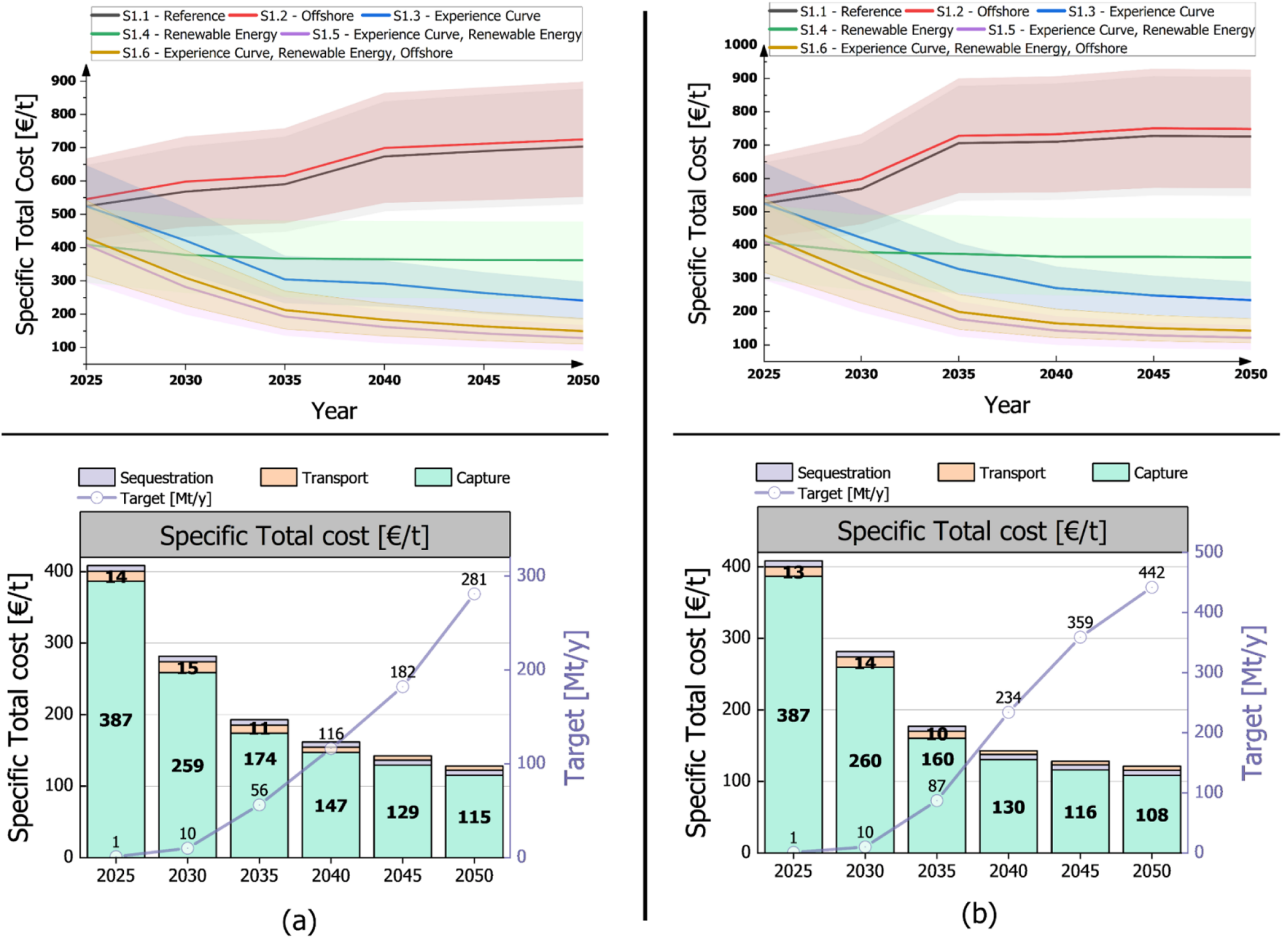
(a) LowDAC scenarios (S1.1–S1.6) and (b) HighDAC scenarios (S2.1–S2.6): The upper section shows the Specific total cost curves for all scenarios (S1.1–S1.6, and S2.1–S2.6) across different periods. The lower section provides a cost breakdown for scenario S1.5 and S2.5, divided into capture, transport, and sequestration components. Additionally, the CO_2_ capture targets in [Mt/y] for each period are also displayed.

Figure 8 illustrates the specific total costs of the DAC supply chain from 2025 to 2050 for the analyzed scenarios. These costs result from optimization under uncertainty, with the shaded areas around each line representing standard deviations. In particular, Figure 8a focuses on LowDAC scenarios (S1.1–S1.6), while Figure 8b presents the HighDAC cases (S2.1–S2.6).

In the LowDAC case (Figure 8a), the most pessimistic scenarios, indicated by the black and red curves (S1.1 and S1.2), assume no capture cost reduction with scale or learning curve, nor a transition of the energy mix to renewable sources. In contrast, the most optimistic one (S1.5), depicted by the purple curve, combines a 12% learning curve and full electrification of DAC systems using renewable energy. This scenario demonstrates a potentially significant reduction in the specific cost of the supply chain, falling to approximately 128 €/t by 2050. As for the HighDAC scenarios (Figure 8b) the results highlight that in the most optimistic scenario (S2.5), which combines a 12% learning curve and full electrification of DAC systems using renewable energy, a significant decrease in costs can be achieved by 2050, down to approximately 121 €/t. Out of this, 108 €/t is attributed to capture costs, as shown in the lower section of Figure 8b. This demonstrates the potentially substantial cost reduction driven by technological advancements and by the integration of renewable energy.

Summarizing, both LowDAC and HighDAC scenarios demonstrate potential cost reductions over time; however, the HighDAC scenarios show a steeper decline in costs due to the larger deployment scale. In both cases, capture costs constitute the majority of the total specific cost, with transport and sequestration costs representing a smaller fraction (around 10% by 2050). As technological advancements and renewable energy adoption increase, the total specific costs decrease significantly. Also, the transition to renewable energy significantly impacts the preference for capture locations as the model approaches 2050. Initially, locations with high GHG emission factors, such as those in Poland and other Eastern and South-Eastern European countries, are less preferred due to their high net CO_2_ removal costs. However, due to the assumed shift to renewable energy, these locations become more favorable. This transition allows for a broader distribution of DAC plants across both Eastern and Northwestern European countries.

### 4.4 Discussion

Understanding the specific characteristics of DAC technologies is fundamental for accurately projecting future costs. The HighDAC scenario highlights how technological advancements and the transition to renewable energy sources can significantly reduce the costs associated with DAC plants. The integration of a 12% learning curve and full electrification of DAC systems using renewable energy in the most optimiztic scenario (S2.5) demonstrates a significant cost reduction, by exhibiting a potential for decrease to approximately 121 €/t by 2050, with 108 €/t attributed to capture costs. However, the results of this study must be contextualized within the broader framework of achieving net-zero emissions targets and on the broader perspective of DAC techno-economics. In fact, while the results are promising, several limitations must be acknowledged. First, if on the one hand the model does not consider the potential cost reductions associated with economies of scale for different DAC plants, which may result in lower estimated costs than what might be achievable with larger-scale operations,^20^ on the flip side the analysis assumes a 1:1 ratio of captured to sequestered CO_2_, without accounting for potential inefficiencies in the DAC plant.^51^ Also, the limited availability of data and information on the energy consumption and productivity of DAC plants^52,53^ led the authors to include in their formulation several assumptions and approximations. While the optimization formulation accounted for uncertainty in these aspects, more accurate and comprehensive data could improve the precision in the projections of the model. Future work could focus on optimizing DAC productivity by investigating advancements in sorbent materials and process efficiencies, which are expected to significantly influence the economics of large-scale DAC deployment. Additionally, examining the implications of land and resource availability, including renewable energy accessibility and spatial constraints, could provide valuable insights into their impact on the feasibility and cost-effectiveness of large-scale DAC systems.

## 5 Conclusions

This study developed a multiperiod mixed-integer linear programming model under uncertainty for the strategic planning of the DAC supply chain at the European level, aiming to minimize overall costs for capture, transport, and geological sequestration of CO_2_. The economic analysis considered the influence of ambient air conditions on sorbent performance, including temperature and humidity impacts on productivity and energy consumption. Seasonal temperature data and interpolated literature values for productivity and energy consumption were used for various European locations, each characterized by different energy costs and GHG emission factors, impacting the net capture cost. Results indicate that with ambitious targets, by implementing learning curves, and a transition to renewable energy, the cost of DAC could fall to around 100 €/t of CO_2_ by 2050. Additionally, offshore sequestration to be reached also with ship transport was shown as a viable option for supporting large-scale DAC deployment in the European framework. The following key takeaways can be summarized:

- The study set up two scenarios, to simulate less (i.e., LowDAC) or more (i.e., HighDAC) ambitious growth rates of DAC chains across Europe.
- In the HighDAC cases, the most pessimistic “business as usual” scenario exhibited a specific total chain cost of 545–748 €/t CO_2_. In contrast, under the most favorable conditions with a 12% learning curve and fully electrified DAC, the total cost was shown to have the potential to fall to 407–121 €/t CO_2_ in the 2025–2050 time frame.
- CO_2_ transport and sequestration together contributed up to 10% of the total DAC cost by 2050. Moreover, while offshore sequestration and ship transport exhibited higher costs with respect to onshore storage and pipeline transport, they demonstrated to offer potentially strategic advantages for locations far from sequestration sites, enhancing the chain flexibility without significantly impacting the total cost of the infrastructure.
- The proximity of DAC installations to sequestration sites is important, but the intrinsic characteristics of the location, such as the local energy mix and costs, have a more significant impact on the optimal selection of capture sites. In fact, countries with a favorable energy mix and low energetic costs, such as those with a high share of renewable energy, are preferred sites for DAC installations. Consequently, this study substantiates that the transition to renewable energy would significantly enhance the cost-effectiveness of DAC plants in those European countries, that are currently characterized by higher GHG emission factors.

This study provides a model for the strategic planning of the DAC supply chain, which can be updated with more accurate data as soon as it becomes available. This flexibility allows for continuous improvement and adaptation of the model to reflect the latest technological advancements and market conditions.

## Supporting Information Available

Input data used for the model (PDF)
