# Supplementary material for: Strategic Design and Multiperiod Optimization under Uncertainty of Solid Sorbent Direct Air Capture Supply Chains in Europe
Source: Ind Eng Chem Res. 2025 Mar 3;64(10):5493–510. doi: 10.1021/acs.iecr.4c04040 (PMC11907705; doi:10.1021/acs.iecr.4c04040)
Supplement: Supplementary file 1 — ie4c04040_si_001.pdf [file ie4c04040_si_001.pdf]

Strategic design and multi-period optimisation under uncertainty of solid sorbent direct air capture supply chains in Europe

Daniel Crîstiu[1], Fengqi You[2], Federico d'Amore[1,*], Fabrizio Bezzo[1,**]

[1] CAPE-Lab - Computer-Aided Process Engineering Laboratory, Department of Industrial Engineering, University of Padova, via Marzolo 9, IT-35131 Padova, Italy.

[2] Robert Frederick Smith School of Chemical and Biomolecular Engineering, Cornell University, Ithaca, NY 14853, USA.

[*] federico.damore@unipd.it

[**] fabrizio.bezzo@unipd.it

Supporting Information

The cost of electricity and thermal energy, based on natural gas prices in each country, is sourced from Eurostat,[1,2] while the greenhouse gas (GHG) emission factors are retrieved from EEA.[3] This study assumes natural gas as the primary source for thermal energy.

Table S1. Input parameters for node n: longitude X_n [rad], latitude Y_n [rad], cost of electric and thermal energy[1,2] in [€/kWh], and GHG emission factor[3] in [gCO_2/kWh].

Node	Location		Country	Cost electric energy [€/kWh]	Cost thermal energy [€/kWh]	GHG emission factor [gCO_2/kWh]
	X_n [rad]	Y_n [rad]				
n1	-0.1476	0.7314	Portugal	0.1394	0.0599	171
n2	-0.1363	0.6932	Portugal	0.1394	0.0599	171
n3	-0.1260	0.6548	Portugal	0.1394	0.0599	171
n4	-0.0960	0.7402	Spain	0.1434	0.0566	178
n5	-0.0863	0.7017	Spain	0.1434	0.0566	178
n6	-0.0776	0.6630	Spain	0.1434	0.0566	178
n7	-0.0436	0.7475	Spain	0.1434	0.0566	178
n8	-0.0357	0.7087	Spain	0.1434	0.0566	178
n9	-0.0286	0.6697	Spain	0.1434	0.0566	178
n10	-0.0446	0.9858	UK	0.3199	0.06876	258
n11	-0.0327	0.9473	UK	0.3199	0.06876	258
n12	-0.0222	0.9087	UK	0.3199	0.06876	258
n13	-0.0047	0.8312	France	0.1939	0.078	74
n14	0.0027	0.7923	France	0.1939	0.078	74
n15	0.0093	0.7533	France	0.1939	0.078	74
n16	0.0153	0.7142	Spain	0.1434	0.0566	178
n17	0.0475	0.8748	France	0.1939	0.078	74
n18	0.0531	0.8357	France	0.1939	0.078	74
n19	0.0582	0.7966	France	0.1939	0.078	74
n20	0.0627	0.7574	France	0.1939	0.078	74
n21	0.0864	1.0734	Norway	0.0559	0.0682	20
n22	0.0921	1.0343	Norway	0.0559	0.0682	20
n23	0.1051	0.9169	Netherlands	0.2128	0.0652	310
n24	0.1085	0.8777	Belgium	0.1542	0.0539	154
n25	0.1114	0.8385	France	0.1939	0.078	74
n26	0.1141	0.7993	France	0.1939	0.078	74
n27	0.1164	0.7600	France	0.1939	0.078	74
n28	0.1681	1.0750	Norway	0.0559	0.0682	20
n29	0.1685	1.0357	Norway	0.0559	0.0682	20
n30	0.1689	0.9964	Denmark	0.1207	0.0582	117
n31	0.1692	0.9572	Denmark	0.1207	0.0582	117
n32	0.1695	0.9180	Germany	0.1784	0.0742	368
n33	0.1697	0.8788	Germany	0.1784	0.0742	368
n34	0.1699	0.8396	Germany	0.1784	0.0742	368
n35	0.1701	0.8003	Italy	0.191	0.062	297
n36	0.1706	0.6820	Italy	0.191	0.062	297
n37	0.2499	1.0739	Sweden	0.0833	0.1276	8
n38	0.2449	1.0347	Sweden	0.0833	0.1276	8
n39	0.2407	0.9955	Sweden	0.0833	0.1276	8
n40	0.2338	0.9172	Germany	0.1784	0.0742	368
n41	0.2310	0.8780	Czech	0.1917	0.0696	407
n42	0.2285	0.8388	Austria	0.2163	0.0613	80
n43	0.2262	0.7996	Italy	0.191	0.062	297
n44	0.2242	0.7603	Italy	0.191	0.062	297

Table S1. (cont'd)

Node	Location		Country	Cost electric energy [€/kWh]	Cost thermal energy [€/kWh]	GHG emission factor [gCO_2/kWh]
	X_n [rad]	Y_n [rad]				
n45	0.2223	0.7209	Italy	0.191	0.062	297
n46	0.3209	1.0312	Sweden	0.0833	0.1276	8
n47	0.3045	0.9533	Poland	0.1483	0.0858	681
n48	0.2979	0.9144	Poland	0.1483	0.0858	681
n49	0.2920	0.8753	Poland	0.1483	0.0858	681
n50	0.2868	0.8363	Austria	0.2163	0.0613	80
n51	0.2821	0.7972	Croatia	0.2105	0.063	170
n52	0.2779	0.7580	Croatia	0.2105	0.063	170
n53	0.2741	0.7187	Italy	0.191	0.062	297
n54	0.2707	0.6793	Italy	0.191	0.062	297
n55	0.2676	0.6398	Italy	0.191	0.062	297
n56	0.4107	1.0639	Finland	0.0853	0.0985	62
n57	0.3827	0.9869	Latvia	0.1508	0.0609	66
n58	0.3714	0.9483	Lithuania	0.1619	0.0549	110
n59	0.3614	0.9096	Poland	0.1483	0.0858	681
n60	0.3526	0.8708	Poland	0.1483	0.0858	681
n61	0.3447	0.8320	Hungary	0.2307	0.0792	181
n62	0.3377	0.7931	Serbia	0.12	0.0517	293
n63	0.3314	0.7540	Serbia	0.12	0.0517	293
n64	0.4886	1.0552	Finland	0.0853	0.0985	62
n65	0.4690	1.0174	Estonia	0.1429	0.0568	693
n66	0.4520	0.9793	Latvia	0.1508	0.0609	66
n67	0.4371	0.9412	Lithuania	0.1619	0.0549	110
n68	0.4020	0.8260	Romania	0.1526	0.0545	250
n69	0.3927	0.7873	Romania	0.1526	0.0545	250
n70	0.3844	0.7486	Serbia	0.12	0.0517	293
n71	0.3768	0.7097	Greece	0.1456	0.0527	394
n72	0.3699	0.6706	Greece	0.1456	0.0527	394
n73	0.4586	0.8182	Romania	0.1526	0.0545	250
n74	0.4472	0.7799	Romania	0.1526	0.0545	250
n75	0.4368	0.7415	Bulgaria	0.1296	0.046	455
n76	0.4190	0.6641	Greece	0.1456	0.0527	394
n77	0.5007	0.7710	Romania	0.1526	0.0545	250
n78	-0.3489	1.1254	Iceland	0.0559	0.0682	8.6
n79	-0.2624	1.1408	Iceland	0.0559	0.0682	8.6
n80	-0.1404	0.9333	Ireland	0.2486	0.0702	305

Table S2. Mean temperature *T* in [°C] and mean relative humidity *RH* in [%] for the 4 different seasons for each location (data taken from Climate Copernicus[4]).

Node	mean *T* [°C]				mean *RH* [%]			
	Winter	Spring	Summer	Autumn	Winter	Spring	Summer	Autumn
n1	8.6	13.8	17.3	10.6	74.7	78.2	75.8	81.4
n2	9.8	16.6	21.2	12.1	76.3	67.0	63.0	76.0
n3	11.6	18.1	24.1	14.3	77.0	67.0	61.6	70.3
n4	6.0	13.7	19.3	8.7	73.3	77.7	75.1	83.2
n5	5.6	14.8	21.6	8.5	69.5	64.6	58.7	81.6
n6	11.7	20.3	27.4	14.6	67.9	60.3	56.7	73.1
n7	5.9	13.1	18.0	8.4	73.6	79.8	81.2	84.0
n8	3.6	12.1	19.2	6.1	75.0	74.8	73.0	84.2
n9	9.3	17.4	24.6	12.2	71.5	70.8	72.1	73.9
n10	3.5	7.4	13.9	9.1	83.6	77.3	77.6	81.6
n11	4.0	9.4	13.1	3.8	83.8	81.6	82.9	83.8
n12	5.2	11.4	14.9	5.1	82.1	81.0	81.9	82.2
n13	6.6	14.5	17.7	7.0	83.0	79.0	80.6	89.9
n14	6.7	15.3	18.9	8.2	81.9	74.5	73.6	87.9
n15	6.2	14.1	19.0	8.3	81.2	78.7	75.7	84.0
n16	10.0	18.1	24.7	13.2	77.1	77.2	77.3	77.1
n17	5.0	12.7	16.3	5.1	86.0	79.5	83.4	88.9
n18	5.4	14.3	17.7	5.8	82.3	77.2	76.7	88.4
n19	4.1	13.2	17.7	5.7	75.9	70.5	69.1	82.7
n20	9.6	17.9	23.2	11.3	76.4	72.8	67.8	76.6
n21	3.3	6.5	14.6	8.8	81.6	76.6	84.3	84.0
n22	2.9	9.4	14.5	5.1	83.2	79.6	73.4	78.2
n23	4.3	12.4	15.7	4.6	82.2	74.7	80.3	86.4
n24	2.4	11.4	14.6	2.5	83.7	74.3	78.4	88.2
n25	2.9	12.3	16.2	3.7	82.4	77.0	80.6	89.9
n26	-2.2	8.7	13.7	0.2	73.9	67.5	66.4	83.2
n27	8.1	15.8	21.6	9.9	72.9	71.0	64.0	75.2
n28	-7.1	4.8	9.2	-5.3	75.8	72.0	76.6	80.9
n29	-3.7	8.3	12.1	-1.2	83.8	72.3	80.8	88.2
n30	2.0	11.0	15.9	4.9	85.7	75.4	81.0	87.3
n31	2.5	11.5	15.6	4.6	86.4	75.9	81.3	87.9
n32	3.1	12.7	16.1	3.9	83.0	74.5	78.5	86.3
n33	1.6	11.9	15.1	2.1	81.5	75.7	79.7	88.9
n34	1.7	13.0	16.2	2.5	81.2	71.5	78.1	87.0
n35	0.5	12.3	17.2	2.6	59.1	60.5	63.0	71.8
n36	10.6	15.3	25.0	18.9	80.0	75.3	63.3	72.0
n37	-8.1	6.3	9.9	-5.4	78.3	71.6	75.0	80.4
n38	-2.6	10.5	14.2	0.9	79.0	71.4	76.7	81.9
n39	-0.8	10.5	13.8	2.2	85.0	77.8	82.1	85.4
n40	2.6	14.7	18.0	4.4	79.1	64.8	68.6	83.2
n41	0.7	12.6	15.9	2.2	77.1	68.2	72.2	82.3
n42	2.0	13.9	17.2	2.8	76.2	64.9	70.1	85.5
n43	5.4	18.3	22.7	7.5	76.4	67.7	71.6	84.1
n44	6.6	17.0	22.9	9.3	72.7	71.4	67.5	77.6
n45	7.8	13.6	23.0	16.3	78.0	72.0	69.0	75.0
n46	-2.3	5.1	14.5	7.3	84.3	71.6	71.0	84.3
n47	0.4	11.6	16.2	4.7	83.5	76.1	81.7	86.9
n48	0.5	13.1	16.6	3.9	81.4	70.1	79.1	87.5
n49	-1.6	11.4	14.4	1.0	85.9	72.7	79.0	89.3
n50	2.2	16.2	19.8	4.9	73.0	64.6	67.2	83.3
n51	3.8	18.1	21.9	7.2	72.6	61.1	62.5	82.7
n52	8.1	14.2	24.3	16.8	63.3	60.0	51.6	65.7
n53	10.2	18.5	25.0	14.3	72.9	73.6	68.2	73.5
n54	7.2	13.1	23.3	16.4	78.8	73.3	65.3	74.0

Table S2. (cont'd)

Node	mean T [°C]				mean RH [%]			
	Winter	Spring	Summer	Autumn	Winter	Spring	Summer	Autumn
n55	11.8	15.8	25.5	20.6	74.0	73.0	64.0	75.0
n56	-5.0	8.1	14.4	2.3	84.1	77.2	79.8	87.0
n57	-3.6	11.1	15.0	2.8	83.1	75.2	80.8	88.3
n58	-2.6	12.6	15.5	3.4	83.3	72.9	81.8	89.1
n59	-1.4	13.7	16.4	3.9	83.2	69.8	80.4	89.6
n60	-1.0	14.0	16.8	3.8	84.5	70.1	78.3	90.4
n61	1.9	17.5	20.8	6.2	81.8	69.1	69.5	88.3
n62	1.6	17.7	21.5	6.7	81.5	67.9	62.8	85.1
n63	-2.0	11.1	16.5	4.2	80.4	76.8	70.0	83.0
n64	-3.5	4.0	16.6	7.2	86.0	75.0	79.0	84.0
n65	-6.6	11.2	14.1	1.1	84.2	76.5	81.0	88.9
n66	-5.8	12.0	14.2	1.7	81.8	70.4	80.0	87.4
n67	-3.9	13.5	15.3	2.5	81.5	69.1	78.8	87.5
n68	-1.5	16.2	19.5	6.8	83.3	69.8	68.3	85.9
n69	-5.5	11.0	15.6	1.2	83.3	73.0	68.0	84.5
n70	0.6	15.8	21.2	6.7	77.9	67.1	57.8	79.6
n71	3.5	16.1	22.5	8.8	76.7	69.0	60.3	79.6
n72	10.4	21.0	27.5	15.6	78.0	73.0	61.3	73.6
n73	-3.9	14.2	17.2	2.9	85.8	73.4	69.6	85.3
n74	1.2	20.7	24.9	8.3	84.1	69.5	60.0	81.2
n75	-1.2	16.5	22.0	6.2	80.4	67.4	54.9	79.0
n76	9.0	15.8	26.7	18.6	77.0	63.0	48.0	67.0
n77	1.3	19.2	23.4	9.6	83.9	68.0	60.0	79.7
n78	-0.1	4.1	8.0	-0.2	78.2	76.9	79.3	81.0
n79	-1.4	1.8	6.1	-1.3	75.5	76.0	83.8	78.8
n80	6.0	10.9	13.3	5.1	86.5	81.1	85.8	88.5

Table S3. Average yearly productivity and electric and thermal energy consumption[5] and the factor *x* reflecting the tons of emitted CO_2 per tons of captured CO_2.

Node	Country	Yearly Productivity [t/module/y]	Yearly electric energy [kWh/tCO_2]	Yearly thermal energy [kWh/tCO_2]	*x* [t emitted CO_2 / t captured CO_2]
n1	Portugal	86.78	314	1331	0.281
n2	Portugal	82.19	338	1473	0.310
n3	Portugal	77.94	363	1598	0.335
n4	Spain	87.84	309	1293	0.285
n5	Spain	85.25	335	1335	0.297
n6	Spain	74.22	395	1686	0.370
n7	Spain	88.26	308	1259	0.279
n8	Spain	87.32	290	1431	0.306
n9	Spain	80.65	353	1529	0.335
n10	UK	90.53	291	1326	0.417
n11	UK	87.68	282	1501	0.460
n12	UK	92.15	287	1128	0.365
n13	France	88.08	295	1266	0.116
n14	France	87.23	302	1314	0.120
n15	France	87.73	304	1292	0.118
n16	Spain	79.60	352	1565	0.341
n17	France	91.01	279	1165	0.107
n18	France	89.91	287	1225	0.112
n19	France	87.24	294	1420	0.127
n20	France	81.40	344	1507	0.137
n21	Norway	90.18	290	1320	0.032
n22	Norway	91.79	285	1306	0.032
n23	Netherlands	88.14	281	1367	0.511
n24	Belgium	86.33	282	1552	0.283
n25	France	83.66	286	1590	0.139
n26	France	88.18	286	1500	0.132
n27	France	84.19	333	1410	0.129
n28	Norway	96.33	270	1377	0.033
n29	Norway	90.34	278	1471	0.035
n30	Denmark	90.01	284	1567	0.217
n31	Denmark	89.81	280	1352	0.191
n32	Germany	83.96	280	1377	0.610
n33	Germany	85.16	284	1569	0.682
n34	Germany	83.37	287	1601	0.695
n35	Italy	81.22	299	1606	0.566
n36	Italy	77.44	364	1623	0.590
n37	Sweden	94.68	272	1407	0.013
n38	Sweden	87.25	282	1535	0.015
n39	Sweden	86.78	282	1529	0.014
n40	Germany	86.33	296	1435	0.637
n41	Czech	83.76	289	1591	0.765
n42	Austria	81.09	294	1629	0.154
n43	Italy	84.68	329	1384	0.509
n44	Italy	83.86	338	1415	0.521
n45	Italy	82.05	341	1486	0.543
n46	Sweden	92.74	283	1275	0.012
n47	Poland	89.40	281	1362	1.119
n48	Poland	82.68	289	1608	1.292
n49	Poland	86.13	283	1551	1.249
n50	Austria	84.39	301	1485	0.143
n51	Croatia	79.69	326	1577	0.323

Table S3. (cont'd)

Node	Country	Yearly Productivity [t/module/y]	Yearly electric energy [kWh/tCO_2]	Yearly thermal energy [kWh/tCO_2]	x [t emitted CO_2 / t captured CO_2]
n52	Croatia	78.50	375	1528	0.323
n53	Italy	78.71	359	1597	0.581
n54	Italy	82.04	344	1476	0.541
n55	Italy	76.04	372	1681	0.610
n56	Finland	88.71	279	1502	0.110
n57	Latvia	85.87	283	1555	0.121
n58	Lithuania	83.89	286	1585	0.206
n59	Poland	82.08	290	1615	1.297
n60	Poland	81.84	290	1625	1.304
n61	Hungary	82.47	302	1538	0.333
n62	Serbia	80.96	316	1559	0.549
n63	Serbia	84.74	286	1577	0.546
n64	Finland	87.50	276	1520	0.111
n65	Estonia	86.26	283	1544	1.266
n66	Latvia	85.48	285	1556	0.122
n67	Lithuania	83.34	288	1595	0.207
n68	Romania	83.65	300	1508	0.452
n69	Romania	85.40	286	1562	0.462
n70	Serbia	81.92	320	1526	0.541
n71	Greece	79.72	330	1582	0.753
n72	Greece	74.92	387	1694	0.820
n73	Romania	81.58	291	1634	0.481
n74	Romania	75.42	351	1677	0.507
n75	Bulgaria	81.13	326	1540	0.849
n76	Greece	76.29	657	1628	0.900
n77	Romania	76.76	340	1653	0.498
n78	Iceland	96.97	267	1360	0.014
n79	Iceland	98.81	252	1549	0.015
n80	Ireland	92.22	278	1111	0.424

Table S4. CO_2 sequestration sites.

Node	Location		Country	Capacity OUT_n^{max} [Mt]	Offshore
	X_n [rad]	Y_n [rad]			
z1	0.1527	0.9817	Denmark	2756	
z2	0.1963	0.9817	Denmark	2340	
z3	0.4145	0.9817	Lithuania	22	
z4	0.4581	0.9817	Lithuanian	15	
z5	-0.0654	0.9381	UK	10850	
z6	0.1527	0.9381	Germany	3164.3	
z7	0.1963	0.9381	Germany	3164.3	
z8	0.24	0.9381	Germany	1714.3	
z9	0.2836	0.9381	Germany	587	
z10	0.3272	0.9381	Poland	587	
z11	0.0654	0.8945	Belgium	199	
z12	0.1091	0.8945	Germany	1090	
z13	0.1527	0.8945	Germany	2804.3	
z14	0.1963	0.8945	Germany	1714.3	
z15	0.24	0.8945	Germany	1714.3	
z16	0.2836	0.8945	Poland	382	
z17	0.3272	0.8945	Poland	1002	
z18	0.3709	0.8945	Poland	382	
z19	0.0218	0.8508	France	1584.4	
z20	0.0654	0.8508	France	2354.4	
z21	0.1091	0.8508	France	1584.4	
z22	0.1963	0.8508	Germany	1714.3	
z23	0.24	0.8508	Austria	766	
z24	0.2836	0.8508	Czech	87	
z25	0.3272	0.8508	Slovak	1716	
z26	0.0654	0.8072	France	1584.4	
z27	0.1527	0.8072	Italy	905	
z28	0.1963	0.8072	Italy	1838.8	
z29	0.24	0.8072	Sloven	94	
z30	0.2836	0.8072	Croatia	2899	
z31	0.3272	0.8072	Hungary	389	
z32	0.3709	0.8072	Romania	4727	
z33	0.4145	0.8072	Romania	4500	
z34	0.5018	0.8072	Bulgaria	2120	
z35	-0.1091	0.7636	Spain	2333.3	
z36	-0.0654	0.7636	Spain	2405.8	
z37	0.1091	0.7636	France	1584.4	
z38	0.24	0.7636	Italy	933.8	
z39	0.3272	0.7636	Bulgaria	197	
z40	-0.0654	0.7199	Spain	2333.3	
z41	0.0218	0.7199	Spain	2405.8	
z42	0.24	0.7199	Italy	933.8	
z43	0.2836	0.7199	Italy	933.8	
z44	0.3709	0.7199	Croatia	390	
z45	-0.1527	0.6763	Portugal	254	
z46	-0.0218	0.6763	Spain	2333.3	
z47	0.1527	0.6763	Italy	71	
z48	0.2836	0.6763	Italy	933.8	
z49	-0.1091	0.6327	Spain	2333.3	
z50	0.0218	1.0254	UK	9790	x
z51	0.0654	1.0254	Norway	9790	x
z52	0.1091	1.0254	Norway	9790	x
z53	0.0218	0.9381	UK	3550	x

Table S5. Ports location.

Node	Location		Port name	Country
	X_n [rad]	Y_n [rad]		
port1	0.0765	0.8939	Antwerpen	Belgium
port2	0.1498	0.9346	Bremerhaven	Germany
port3	0.1744	0.9346	Hamburg	Germany
port4	0.1424	0.9344	Wilhelmshaven	Germany
port5	0.4323	1.0374	Tallinn	Estonia
port6	0.4125	0.6624	Peiraeus	Greece
port7	-0.0951	0.6306	Algeciras	Spain
port8	0.0378	0.7216	Barcelona	Spain
port9	-0.0056	0.6885	Valencia	Spain
port10	0.0415	0.8907	Dunkerque	France
port11	0.0027	0.8634	Le Havre	France
port12	0.0937	0.7556	Marseille	France
port13	0.1557	0.7749	Genova	Italy
port14	0.3005	0.7065	Taranto	Italy
port15	0.2402	0.7969	Trieste	Italy
port16	0.4207	0.9947	Riga	Latvia
port17	0.0839	0.9148	Amsterdam	Netherlands
port18	0.0748	0.9056	Rotterdam	Netherlands
port19	-0.1548	0.6624	Sines	Portugal
port20	0.4998	0.7710	Constanta	Romania
port21	0.2082	1.0071	Goteborg	Sweden
port22	-0.0033	0.9360	Immingham	UK
port23	0.0087	0.8988	London	UK
port24	-0.0879	0.9026	Milford Haven	UK
port25	-0.0244	0.8883	Southampton	UK
port26	-0.0202	0.9530	Tees Hartlepool	UK
port27	0.0926	1.0540	Bergen	Norway
port28	-0.0586	0.8334	Lorient	France
port29	-0.0988	0.7598	Gijon	Spain
port30	0.1589	0.6844	Cagliari	Italy
port31	0.2655	0.6492	Augusta	Italy
port32	-0.3831	1.1192	Reykjavík	Iceland
port33	-0.1087	0.9310	Dublin	Irlanda

Renewable electricity prices over the periods *t* are estimated from Sievert et al.[6] using their cost projections. Table S6 displays cost projections from 2022 to 2050, with the first projection for 2025 being retrieved from IRENA.[7]

Table S6. Renewable electricity cost projections.

Period	2025	2030	2035	2040	2045	2050
Cost [€/kWh]	0.049	0.026	0.021	0.019	0.018	0.017

Supplementary bibliography

(1) Eurostat. Electricity prices for non-household consumers. 2024. https://ec.europa.eu/eurostat/databrowser/view/nrg_pc_205/default/table?lang=en

(2) Eurostat. Gas prices for non-household consumers. 2024. https://ec.europa.eu/eurostat/databrowser/view/nrg_pc_203/default/table?lang=en

(3) EEA. Greenhouse gas emission intensity of electricity generation. 2024. https://www.eea.europa.eu/en/analysis/indicators/greenhouse-gas-emission-intensity-of-1

(4) Climate Copernicus. European State of the Climate. 2024. https://climate.copernicus.eu/

(5) Wiegner, J.F.; Grimm, A.; Weimann, L.; Gazzani, M. Optimal Design and Operation of Solid Sorbent Direct Air Capture Processes at Varying Ambient Conditions. Ind. Eng. Chem. Res. 2022, 61, 12649-12667. https://doi.org/10.1021/acs.iecr.2c00681

(6) Sievert, K.; Schmidt, T.S.; Steffen, B. Considering technology characteristics to project future costs of direct air capture. Joule 2024, 8, 979-999. https://doi.org/10.1016/j.joule.2024.02.005

(7) IRENA. Renewable energy statistics. 2023. https://www.irena.org/-/media/Files/IRENA/Agency/Publication/2023/Jul/IRENA_Renewable_energy_statistics_2023.pdf?rev=7b2f44c294b84cad9a27fc24949d2134
